# Real-World Treatment Patterns and Determinants of Therapy in Pediatric Atopic Dermatitis: A 10-Year Experience from a Tertiary Referral Center in Thailand

**DOI:** 10.3390/children13030385

**Published:** 2026-03-09

**Authors:** Nuttida Yusakda, Leelawadee Techasatian, Piyadarat Asawasakulchokedee, Rattapon Uppala, Phanthila Sitthikarnkha, Suchaorn Saengnipanthkul, Prapassara Sirikarn, Pope Kosalaraksa

**Affiliations:** 1 Department of Pediatrics, Faculty of Medicine, Khon Kaen University, Khon Kaen 40000, Thailandpiyaas@kku.ac.th (P.A.);; 2 Department of Epidemiology and Biostatistics, Faculty of Public Health, Khon Kaen University, Khon Kaen 40000, Thailand

**Keywords:** atopic dermatitis, treatment patterns, children

## Abstract

**Highlights:**

**What are the main findings?**

- Antihistamines and moderate-potency topical corticosteroids were the most commonly prescribed treatments for pediatric atopic dermatitis.
- The use of topical non-steroidal agents and biologic therapies remained limited over the 10-year study period.
- Adolescents were less likely to receive topical non-steroidal medications than infants.
- Healthcare coverage, particularly under the Civil Servant Medical Benefit Scheme, strongly influenced access to non-steroidal therapies.

**What are the implications of the main findings?**

- Pediatric atopic dermatitis management in Thailand remains dominated by conventional therapies.
- Socioeconomic and insurance-related factors contribute to unequal access to advanced treatments.
- Improved reimbursement policies and treatment accessibility may enhance equity in pediatric AD care.
- Targeted education for healthcare providers and families may support more appropriate and guideline-based treatment use.

**Abstract:**

Background: Atopic dermatitis (AD) is a chronic inflammatory skin disease in children requiring long-term management, yet real-world data on treatment patterns remain limited. Objective: To evaluate treatment trends and factors associated with topical non-steroidal medication use in pediatric AD. Methods: We retrospectively analyzed 3982 children with AD treated at a tertiary referral center in Thailand between 2015 and 2024. Demographic data, healthcare coverage, and prescribed treatments were reviewed. Multivariable logistic regression was used to identify factors associated with topical non-steroidal use. Results: The median age was 7 years, with no sex difference. The most commonly prescribed treatments were antihistamines (75.0%), topical corticosteroids (47.6%), moisturizers (43.9%), systemic immunosuppressants (15.7%), topical non-steroidal agents including topical calcineurin inhibitors, phosphodiesterase-4 inhibitors, and Janus kinase (JAK) inhibitors (12.7%), and biologics (0.1%). Moderate-potency corticosteroids predominated. Adolescents were less likely to receive topical non-steroidal agents than infants (OR 0.66, 95% CI 0.50–0.87), whereas patients under the Civil Servant Medical Benefit Scheme (CSMBS) had higher access than those under the Universal Coverage Scheme (UCS) (OR 8.40, 95% CI 5.76–12.25). Conclusions: Pediatric AD management was dominated by conventional therapies, with limited access to advanced treatments. Age and healthcare coverage strongly influenced prescribing patterns, highlighting the need for more equitable access.

## 1. Introduction

Atopic dermatitis (AD) is a common chronic inflammatory skin disease, affecting up to 20% of children worldwide and substantially impairing quality of life. The disease often begins in early childhood and follows a relapsing course that requires long-term management.

Standard treatment of AD includes patient education, basic skin care, regular use of moisturizers, and avoidance of triggering factors [1]. Topical corticosteroids (TCSs) remain the mainstay of pharmacological therapy and are recommended as first-line treatment in most clinical guidelines. However, concerns regarding adverse effects and steroid phobia among patients and caregivers [2,3] have contributed to increasing interest in topical non-steroidal alternatives, including topical calcineurin inhibitors, phosphodiesterase-4 inhibitors, and Janus kinase (JAK) inhibitors. For patients with moderate-to-severe atopic dermatitis or those with an inadequate response to topical therapy, escalation to systemic treatment is often required. Conventional systemic immunosuppressive agents, including methotrexate and cyclosporine, have long been used to control severe disease [4]. More recently, advances in targeted therapies have expanded treatment options, including biologic agents [4] targeting interleukin (IL)-4 and IL-13 pathways [5] and oral JAK inhibitors. These newer biologic and targeted therapies have demonstrated favorable efficacy and safety profiles in both adult and pediatric populations [6], leading to their approval for the management of moderate-to-severe atopic dermatitis in children and adolescents in several regions [7,8,9,10,11]. However, access to these therapies remains highly dependent on healthcare coverage, reimbursement policies, and national healthcare systems, which may substantially influence real-world treatment patterns.

In Thailand, a wide range of therapies for AD is available; however, access to advanced treatments varies according to healthcare coverage and institutional resources. Real-world prescribing practices may therefore differ from guideline recommendations and international experience. Despite the high burden of pediatric AD, data on real-world treatment patterns and factors influencing therapeutic choices in children remain limited, particularly in Southeast Asia. This study aimed to evaluate medication use patterns and determinants of therapy among pediatric patients with AD in a university hospital setting, where comprehensive treatment options are available. Understanding these patterns may support more appropriate, accessible, and cost-effective management strategies for children with AD.

### Objectives

This study aimed to describe real-world treatment patterns and temporal trends in pediatric patients with atopic dermatitis treated at a tertiary referral center over a 10-year period. In particular, focusing on the utilization of topical non-steroidal medications across different age groups. Furthermore, we aimed to find out the demographic and healthcare-related characteristics linked to the application of topical non-steroidal medications including pimecrolimus, tacrolimus, and the phosphodiesterase-4 (PDE-4) inhibitor (crisaborole), which are the agents currently available and used for atopic dermatitis management in Thailand, in comparison to alternative treatment modalities.

## 2. Materials and Methods

### 2.1. Methods

#### 2.1.1. Study Design and Participants

A retrospective cross-sectional study was conducted at the Department of Pediatrics, Faculty of Medicine, Khon Kaen University, Thailand, using electronic medical record data from the hospital information system (Health Object program) between 1 January 2015 and 30 December 2024. Treatment exposure was defined as the first recorded prescription for each patient during the study period, with repeated prescriptions counted only once. Analyses were therefore conducted at the patient level, and temporal changes in prescribing patterns and within-patient correlations were not evaluated.

#### 2.1.2. Statistical Analysis

The characteristics of participants were reported as frequencies and percentages for categorical data. Continuous data were presented using the mean with standard deviation (SD) and the median with minimum and maximum values.

A generalized linear model (GLM) with a binomial distribution was implemented. The GLM utilized the logit link function to report the odds ratio (OR) with 95% CI, and then utilized the identity link function to report the difference with 95% CI. All analyses were performed using StataNow, version 19.5.

## 3. Results

### 3.1. Patient Characteristics

A total of 3982 children with atopic dermatitis were included in the analysis. Baseline demographic and clinical characteristics are presented in Table 1. Of these, 1958 (49.2%) were male and 2024 (50.8%) were female. The mean age at diagnosis was 7.77 ± 5.86 years, with a median age of 7 years (range, 0–18 years).

Patients were categorized into four age groups: infants: <1 year (19.7%), pre-school age: 1.1–5 years (23.3%), school age: 5.1–11 years (27.2%), and adolescent: 11.1–18 years (29.8%), with adolescents comprising the largest proportion. Regarding healthcare coverage, 39.4% were covered by the Civil Servant Medical Benefit Scheme (CSMBS), 35.0% were self-pay, 24.5% were under the Universal Coverage Scheme (UCS), and 1.1% had private insurance.

### 3.2. Treatment Patterns by Age Group

Medication use according to age group is summarized in Table 2. Antihistamines were the most commonly prescribed medications, used in 2985 patients (75.0%), with the highest use observed in pre-school age children (80.7%) and adolescents (80.4%), Table 2.

Oral antibiotics were prescribed in 632 patients (15.9%), with the greatest use in the pre-school age group (20.7%). Topical corticosteroids were used in 1894 patients (47.6%). Moderate-potency corticosteroids were most frequently prescribed (36.1%), followed by low-potency (14.2%) and high-potency agents (3.4%), respectively.

The use of high-potency corticosteroids increased with age, from 0.5% in infants to 4.8% in adolescents, whereas low-potency corticosteroids were most commonly used in infants (38.7%) and declined with age, Table 3.

Topical non-steroidal medications were prescribed in 505 patients (12.7%), with similar use across age groups (10.1–15.1%). Systemic immunosuppressive agents were used in 625 patients (15.7%), with increasing use from infancy (9.0%) to adolescence (19.0%). Biologic therapy was rarely prescribed, with only five patients (0.1%) receiving these agents (Table 2).

Moisturizers were prescribed in 1746 patients (43.9%). Plain moisturizers were used in 28.0% of patients, while moisturizer-plus products were used in 19.9%. The use of enhanced moisturizers increased with age, reaching 23.9% in adolescents.

**Figure 1:**
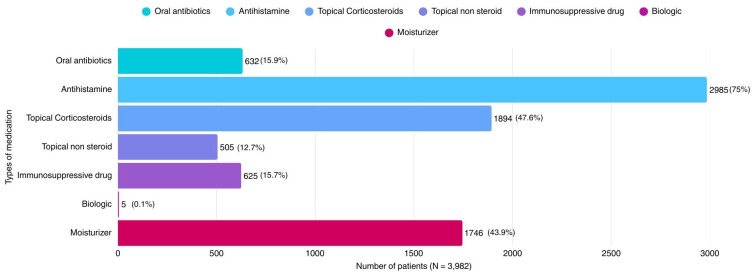
Distribution of pharmacologic treatments prescribed for pediatric atopic dermatitis in the study cohort (N = 3982). Bars represent the number and percentage of patients receiving each treatment category during the study period. Patients may have received more than one type of medication.

Overall treatment patterns during the study period are illustrated in Figure 1. Antihistamines and topical corticosteroids remained the mainstay of treatment from 2015 to 2024. In contrast, the use of systemic immunosuppressive agents and topical non-steroidal medications gradually increased over time, whereas biologic use remained minimal in our study population.

### 3.3. Factors Associated with Topical Non-Steroidal Medication Use

Factors associated with topical non-steroidal medication use are shown in Table 4. In multivariable logistic regression analysis, sex was not significantly associated with medication use (female vs. male: OR 1.04, 95% CI 0.86–1.25, *p* = 0.681). Compared with infants, adolescents were significantly less likely to receive topical non-steroidal medications (OR 0.66, 95% CI 0.50–0.87, *p* = 0.003).

Healthcare coverage was strongly associated with prescribing patterns. Compared with UCS, patients covered by CSMBS were more likely to receive topical non-steroidal medications (OR 8.40, 95% CI 5.76–12.25, *p* < 0.001). Self-pay patients also had higher odds of receiving these medications (OR 3.16, 95% CI 2.12–4.71, *p* < 0.001). Private insurance coverage was not significantly associated with medication use.

## 4. Discussion

This study provides comprehensive real-world evidence on treatment patterns among pediatric patients with atopic dermatitis (AD) in a tertiary referral center in Thailand over a 10-year period. Our findings demonstrate that moderate-potency topical corticosteroids remain the most frequently prescribed agents across all age groups, while the use of high-potency corticosteroids and systemic immunosuppressive therapies increases with age. In addition, healthcare coverage was a major determinant of access to topical non-steroidal therapies, highlighting important disparities in treatment availability.

Consistent with current international guidelines [12,13,14], topical corticosteroids constituted the cornerstone of treatment in our cohort, with moderate-potency agents being most commonly used across all age groups. Low-potency corticosteroids were preferentially prescribed in infants, whereas high-potency agents were more frequently used in older children and adolescents. This age-related trend likely reflects increasing disease severity, chronicity, and treatment resistance in older patients, as well as greater tolerance of potential adverse effects. Similar prescribing patterns have been reported in previous observational studies from Europe and Asia [15,16,17].

Systemic immunosuppressive agents, including oral prednisolone, methotrexate, and cyclosporin A, were prescribed more frequently in adolescents than in younger children. This finding is consistent with earlier reports indicating that moderate-to-severe AD often emerges during late childhood and adolescence and may require systemic therapy for adequate disease control [7,8,9,12,18]. However, physicians’ decisions to initiate systemic treatment are influenced not only by disease severity but also by treatment adherence, relapse frequency, quality of life impairment, and psychosocial burden.

Despite increasing global availability of biologic and targeted therapies for pediatric AD [19] with approval for moderate-to-severe atopic dermatitis [7,11], their use in our study population was extremely limited, with only 0.1% of patients receiving biologic treatment. This low utilization rate likely reflects restricted access, high cost, and limited reimbursement within the Thai healthcare system. In addition, structural and regulatory factors may contribute to the limited use of biologic therapies. Biologic agents for atopic dermatitis have only recently been approved in Thailand, while the present study includes retrospective data spanning the past 10 years; therefore, the observed number of biologic prescriptions remains low and may not fully reflect current or future utilization. Access may also be limited by pediatric age restrictions and reimbursement policies across different healthcare coverage schemes. Furthermore, eligibility criteria related to disease severity and prior treatment failure may further restrict the use of biologics in routine clinical practice. Similar barriers have been reported in other low- and middle-income countries [15]. These illustrate our findings and highlight the possible implications of healthcare coverage limitations on options for treatment. Expanding access to these advanced therapies represents an important future goal for improving equity in AD care.

Our findings also demonstrate that healthcare coverage plays a critical role in determining treatment options [16,20]. Patients covered under the CSMBS had significantly higher odds of receiving topical non-steroidal medications compared with those under the UCS. This disparity reflects differences in reimbursement policies, as CSMBS provides full reimbursement and free access to higher-cost medications, including topical non-steroidal agents, whereas patients covered by the UCS have more restricted access and often must pay out of pocket for expensive therapies. In contrast, self-pay patients represent a subgroup with greater willingness and financial ability to afford these medications. Consequently, families under UCS or without comprehensive coverage may face restricted treatment choices, potentially leading to suboptimal disease control.

Steroid phobia remains an important contributor to treatment nonadherence in pediatric AD. Parental concerns regarding the adverse effects of topical corticosteroids can negatively influence treatment adherence and outcomes [1,2,3,21]. In our study, some families chose to pay out of pocket or use private insurance to obtain non-steroidal alternatives. These findings emphasize the need for effective patient and caregiver education to address misconceptions about corticosteroid safety and to promote evidence-based treatment practices.

Oral antihistamines were prescribed in approximately 75% of patients, reflecting their widespread use for pruritus relief and management of associated allergic comorbidities [22]. Although antihistamines have limited direct effects on skin inflammation and are not routinely recommended for atopic dermatitis in current guidelines, they are sometimes used in clinical practice, particularly sedating agents to help manage nocturnal pruritus and sleep disturbance. The high prescribing rate observed in this study may also reflect the tertiary care setting, where patients often present with more persistent or difficult-to-control symptoms and clinicians may employ adjunctive therapies for symptomatic relief. In addition, antihistamines may have been prescribed for coexisting atopic conditions, such as allergic rhinitis or urticaria. However, the electronic health record data did not consistently capture the specific indication, type of antihistamine, or treatment duration. Therefore, this pattern likely reflects real-world clinical practice rather than strictly guideline-directed therapy, while also highlighting the importance of individualized prescribing.

Moisturizers remain a fundamental component of AD management by restoring skin barrier function and preventing disease exacerbations [13,14,23,24]. In our cohort, moisturizer use ranged from 42.3% to 46.8% across age groups. This relatively moderate utilization may be influenced by the wide availability of over-the-counter products and caregiver preferences. In addition, climatic factors in tropical countries such as Thailand may reduce compliance with thick or greasy emollients due to discomfort, sweating, and cosmetic concerns [1,21]. These factors may contribute to inconsistent application and suboptimal barrier repair.

Beyond physical symptoms, AD imposes substantial psychological and social burdens on affected children and their families [25,26]. Caregivers frequently experience emotional stress, sleep disturbance, and financial strain, while children may face stigmatization, bullying, and impaired peer relationships. Poorly controlled disease has been associated with increased risks of anxiety and depression extending into adulthood. These psychosocial aspects are often underrecognized in routine clinical practice and warrant greater attention in comprehensive care models.

Our multivariable analysis identified age and healthcare coverage as factors associated with the use of topical non-steroidal medications. Adolescents were less likely than infants to receive non-steroidal therapies, which may reflect differences in treatment approaches across age groups. However, in the absence of detailed severity data, this observation should be interpreted cautiously. Alternative explanations may include referral bias in a tertiary care setting, treatment escalation practices in older children, differences in caregiver preferences by age, and regulatory restrictions for certain medications in younger children. In addition, patients under CSMBS and self-pay schemes were substantially more likely to receive non-steroidal medications, suggesting that financial and institutional factors may influence prescribing patterns. These findings highlight the complex interplay between clinical considerations, healthcare policy, and socioeconomic context in shaping treatment patterns in pediatric atopic dermatitis.

### Limitations

This study has several limitations. First, the retrospective design is subject to incomplete documentation and potential residual confounding. Several clinically important variables that may influence treatment decisions—such as disease severity (e.g., EASI, SCORAD, or IGA scores), anatomical distribution, comorbid atopic diseases, prior treatment failure, and physician specialty—were not consistently recorded in the electronic medical records and therefore could not be included in the multivariable analysis. As a result, the adjusted model was limited to variables reliably captured in the database (age, sex, and healthcare coverage). The absence of these factors may introduce residual confounding and could overestimate the observed association between healthcare coverage and prescribing patterns. Second, data were derived from a single tertiary referral center, which may introduce referral bias and limit generalizability to other care settings. Nevertheless, the large sample size and extended 10-year study period provide valuable insight into real-world prescribing patterns in pediatric atopic dermatitis.

## 5. Conclusions

In this large real-world retrospective cross-sectional study of pediatric patients with atopic dermatitis treated at a tertiary referral center in Thailand, moderate-potency topical corticosteroids were the most commonly used therapies, while the use of topical non-steroidal agents and biologic treatments remained limited. Access to non-steroidal treatments was associated with patient age and healthcare coverage. However, these findings should be interpreted cautiously, as the retrospective design and the absence of key clinical variables—such as disease severity and other factors influencing treatment decisions—introduce a high risk of residual confounding. Therefore, the observed relationships should be considered associations rather than determinants of prescribing patterns. Despite these limitations, the results highlight potential disparities in treatment availability and suggest the need for policies and strategies that promote more equitable access to advanced therapies.

## Figures and Tables

**Table 1:** Demographic characteristics, age at diagnosis, and primary healthcare coverage among pediatric patients with atopic dermatitis included in the study cohort (n = 3982).

Characteristics	n	%
Sex		
Male	1958	49.2%
Female	2024	50.8%
Age at first diagnosis (years)		
≤1	785	19.7%
1.1–5	927	23.3%
5.1–11	1083	27.2%
11.1–18	1187	29.8%
Mean (SD)	7.77	(5.86)
Median (Min:Max)	7	(0:18)
Current primary payor		
UCS	974	24.5%
CSMBS	1571	39.4%
Self-pay	1393	35.0%
Insurance	44	1.1%

CSMBS: Civil Servant Medical Benefit Scheme; UCS: Universal Coverage Scheme.

**Table 2:** Age-stratified use of topical and systemic therapies among pediatric patients with atopic dermatitis in a tertiary-care setting. Data are presented as numbers (percentages) within each age group; multiple treatments per patient were permitted (n = 3982).

Characteristics	Age Group				Total (n = 3982)
	≤1 (n = 785)	1.1–5 (n = 927)	5.1–11 (n = 1083)	11.1–18 (n = 1187)	
	n	n	n	n	n
Oral Antibiotics	115 (14.7%)	192 (20.7%)	190 (17.5%)	135 (11.4%)	632 (15.9%)
Antihistamine	437 (55.7%)	748 (80.7%)	846 (78.1%)	954 (80.4%)	2985 (75.0%)
Topical Corticosteroids	596 (75.9%)	482 (52.0%)	453 (41.8%)	363 (30.6%)	1894 (47.6%)
Systemic immune suppressive drug	71 (9.0%)	136 (14.6%)	192 (17.7%)	226 (19.0%)	625 (15.7%)
Biologic	1 (0.1%)	0(0.0%)	4 (0.4%)	0(0.0%)	5 (0.1%)
Topical Non-steroid Medication: (Pimecrolimus, Tacrolimus, Crisaborole)					
	114 (14.5%)	108 (11.6%)	163 (15.1%)	120 (10.1%)	505 (12.7%)
Moisturizer					
Plain	229 (29.2%)	282 (30.4%)	338 (31.2%)	265 (22.3%)	1114 (28.0%)
Moisturizer Plus	134 (17.1%)	159 (17.2%)	217 (20.0%)	284 (23.9%)	794 (19.9%)
Total	332 (42.3%)	397 (42.8%)	507 (46.8%)	510 (43.0%)	1746 (43.9%)

**Table 3:** Distribution of topical corticosteroid potency by age group among pediatric patients with atopic dermatitis.

Potency of Topical Corticosteroids	Age Group								Total (n = 1894)	
	≤1 (n = 596)		1.1–5 (n = 482)		5.1–11 (n = 453)		11.1–18 (n = 363)			
	n	%	n	%	n	%	n	%	n	%
High Potency	4	0.5%	20	2.2%	54	5.0%	57	4.8%	135	3.4%
Moderate Potency	381	48.5%	378	40.8%	369	34.1%	309	26%	1437	36.1%
Low Potency	304	38.7%	156	16.8%	79	7.3%	28	2.4%	567	14.2%

**Table 4:** Factors associated with topical non-steroid medication.

Factors	Topical Non-Steroid Medication (n = 505)		OR	95% CI	*p*-Value
	n	%			
Sex					
Male	244	48.3%	1.000		
Female	261	51.7%	1.040	0.863 to 1.253	0.681
Age at first diagnosis (years)					
≤1	114	22.6%	1.000		
1.1–5	108	21.4%	0.776	0.585 to 1.029	0.079
5.1–11	163	32.3%	1.043	0.805 to 1.351	0.751
11.1–18	120	23.7%	0.662	0.503 to 0.871	0.003
Current primary payor					
UCS	31	6.1%	1.000		
CSMBS	340	67.3%	8.402	5.761 to 12.254	<0.001
Self-pay	131	25.9%	3.158	2.116 to 4.713	<0.001
Insurance	3	0.6%	2.226	0.653 to 7.582	0.201

## Data Availability

Data availability upon request with corresponding author.

## References

[1] Nummak P., Techasatian L., Uppala R., Sitthikarnkha P., Saengnipanthkul S., Sirikarn P. (2024). Parental Attitudes and Practices regarding Atopic Dermatitis: A Cross-Sectional Study among a Thai Population. Children.

[2] Promthes T., Techasatian L., Salee-Or S., Uppala R., Sitthikarnkha P., Saengnipanthkul S., Sirikarn P., Kosalaraksa P. (2025). Topical corticosteroid phobia among caregivers: A study in atopic and nonatopic dermatitis children by using the TOPICOP score. Skin. Health Dis..

[3] Tangthanapalakul A., Chantawarangul K., Wananukul S., Tempark T., Chatproedprai S. (2023). Topical corticosteroid phobia in adolescents with eczema and caregivers of children and adolescents with eczema: A cross-sectional survey. Pediatr. Dermatol..

[4] Hamza Osman S.K., Mohamed Ahmed M.A., Idrees H., Mohammad Ali A.M.H., Ahmed Taha A.H., Musa Shaikhelsafi F.H., Mirghani Hamour A.M. (2025). Systemic Therapies for Moderate-to-Severe Atopic Dermatitis in Children and Adolescents: A Systematic Review. Cureus.

[5] Gargiulo L., Ferrucci S.M., Ibba L., Ingurgio R.C., Alfano A., Amoruso G.F., Balato A., Barbagallo T., Barei F., Bellinato F. (2025). Five-year drug survival of dupilumab in atopic dermatitis: Italian landscape AD real-world study. J. Eur. Acad. Dermatol. Venereol..

[6] Li K.H., Duffy E.K., Luong J., Devine B. (2026). Biologic Treatments in Adolescents with Moderate-to-Severe Atopic Dermatitis: A Systematic Literature Review and Network Meta-analysis. Ann. Pharmacother..

[7] Chu A.W.L., Wong M.M., Rayner D.G., Guyatt G.H., Díaz Martinez J.P., Ceccacci R., Zhao I.X., McMullen E., Srivastava A., Wang J. (2023). Systemic treatments for atopic dermatitis (eczema): Systematic review and network meta-analysis of randomized trials. J. Allergy Clin. Immunol..

[8] Eichenfield L.F., Flohr C., Sidbury R., Siegfried E., Szalai Z., Galus R., Yao Z., Takahashi H., Barbarot S., Feeney C. (2021). Efficacy and Safety of Abrocitinib in Combination with Topical Therapy in Adolescents with Moderate-to-Severe Atopic Dermatitis: The JADE TEEN Randomized Clinical Trial. JAMA Dermatol..

[9] Simpson E.L., Paller A.S., Siegfried E.C., Boguniewicz M., Sher L., Gooderham M.J., Beck L.A., Guttman-Yassky E., Pariser D., Blauvelt A. (2020). Efficacy and Safety of Dupilumab in Adolescents with Uncontrolled Moderate to Severe Atopic Dermatitis: A Phase 3 Randomized Clinical Trial. JAMA Dermatol..

[10] Lin T.-L., Fan Y.-H., Fan K.-S., Juan C.-K., Chen Y.-J., Wu Y.-J.C. (2024). Reduced atopic march risk in pediatric atopic dermatitis patients prescribed dupilumab versus conventional immunomodulatory therapy: A population-based cohort study. J. Am. Acad. Dermatol..

[11] Paller A.S., Marcoux D., Ramien M., Baselga E., Carvalho V.O., Ardusso L.R.F., de Graaf M., Pasmans S., Toledo-Bahena M., Rubin C. (2025). Systemic Treatments in Moderate-to-Severe Atopic Dermatitis in Pediatric Patients up to 12 Years of Age: Real-World Treatment Outcomes from the PEDISTAD Registry. Am. J. Clin. Dermatol..

[12] Eichenfield L.F., Tom W.L., Berger T.G., Krol A., Paller A.S., Schwarzenberger K., Bergman J.N., Chamlin S.L., Cohen D.E., Cooper K.D. (2014). Guidelines of care for the management of atopic dermatitis: Section 2. Management and treatment of atopic dermatitis with topical therapies. J. Am. Acad. Dermatol..

[13] Wollenberg A., Kinberger M., Arents B., Aszodi N., Barbarot S., Bieber T., Brough H.A., Calzavara-Pinton P., Christen-Zaech S., Deleuran M. (2025). European Guideline (EuroGuiDerm) on atopic eczema: Living update. J. Eur. Acad. Dermatol. Venereol..

[14] Chu D.K., Schneider L., Asiniwasis R.N., Boguniewicz M., De Benedetto A., Ellison K., Frazier W.T., Greenhawt M., Huynh J., AAAAI/ACAAI JTF Atopic Dermatitis Guideline Panel (2024). Atopic dermatitis (eczema) guidelines: 2023 American Academy of Allergy, Asthma and Immunology/American College of Allergy, Asthma and Immunology Joint Task Force on Practice Parameters GRADE- and Institute of Medicine-based recommendations. Ann. Allergy Asthma Immunol..

[15] Mohn C.H., Blix H.S., Brænd A.M., Nafstad P., Nygard S., Halvorsen J.A. (2022). Treatment Patterns of Atopic Dermatitis Medication in 0–10-Year-Olds: A Nationwide Prescription-Based Study. Dermatol Ther.

[16] Paller A.S., Siegfried E.C., Vekeman F., Gadkari A., Kaur M., Mallya U.G., Héroux J., Miao R., Mina-Osorio P. (2020). Treatment patterns of pediatric patients with atopic dermatitis: A claims data analysis. J. Am. Acad. Dermatol..

[17] Lee J.H., Choi A., Noh Y., Oh I.-S., Jeon J.-Y., Yoo H.-J., Shin J.-Y., Son S.W. (2022). Real-world treatment patterns for atopic dermatitis in South Korea. Sci. Rep..

[18] Davis D.M.R., Drucker A.M., Alikhan A., Bercovitch L., Cohen D.E., Darr J.M., Eichenfield L.F., Frazer-Green L., Paller A.S., Schwarzenberger K. (2024). Guidelines of care for the management of atopic dermatitis in adults with phototherapy and systemic therapies. J. Am. Acad. Dermatol..

[19] Gargiulo L., Vignoli C.A., Ibba L., Cortese A., Valenti M., Costanzo A., Narcisi A. (2023). Real-life effectiveness and safety of dupilumab in adolescents with atopic dermatitis: A 52-week single-center retrospective study. J. Dermatol. Treat..

[20] Skinner A.C., Mayer M.L. (2007). Effects of insurance status on children’s access to specialty care: A systematic review of the literature. BMC Health Serv. Res..

[21] Nitiyarom R., Banomyong N., Wisuthsarewong W. (2022). Knowledge about, attitude toward, and practices in skin care among Thai adolescents. J. Cosmet. Dermatol..

[22] Chunharas A., Wisuthsarewong W., Wananukul S., Viravan S. (2002). Therapeutic efficacy and safety of loratadine syrup in childhood atopic dermatitis treated with mometasone furoate 0.1 per cent cream. J. Med. Assoc. Thail. Chomaihet Thangphaet..

[23] Techasatian L., Kiatchoosakun P. (2022). Effects of an emollient application on newborn skin from birth for prevention of atopic dermatitis: A randomized controlled study in Thai neonates. J. Eur. Acad. Dermatol. Venereol..

[24] Bradshaw L.E., Wyatt L.A., Brown S.J., Haines R.H., Montgomery A.A., Perkin M.R., Lawton S., Sach T.H., Chalmers J.R., Ridd M.J. (2023). Emollients for prevention of atopic dermatitis: 5-year findings from the BEEP randomized trial. Allergy.

[25] Otsuka A., Wang C., Torisu-Itakura H., Matsuo T., Isaka Y., Anderson P., Piercy J., Austin J., Marwaha S., Tanaka A. (2024). Patient and family burden in pediatric atopic dermatitis and its treatment pattern in Japan. Int. J. Dermatol..

[26] Paller A.S., Rangel S.M., Chamlin S.L., Hajek A., Phan S., Hogeling M., Castelo-Soccio L., Lara-Corrales I., Arkin L., Lawley L.P. (2024). Stigmatization and Mental Health Impact of Chronic Pediatric Skin Disorders. JAMA Dermatol..

